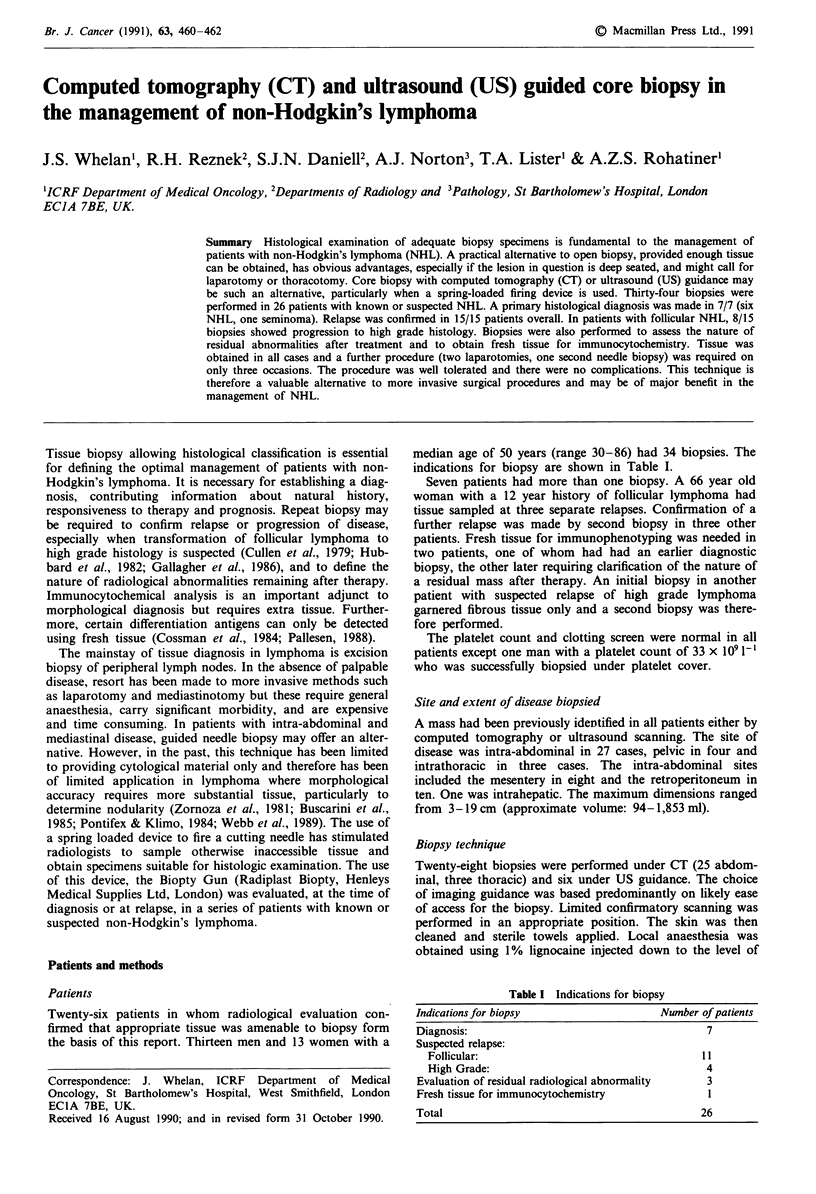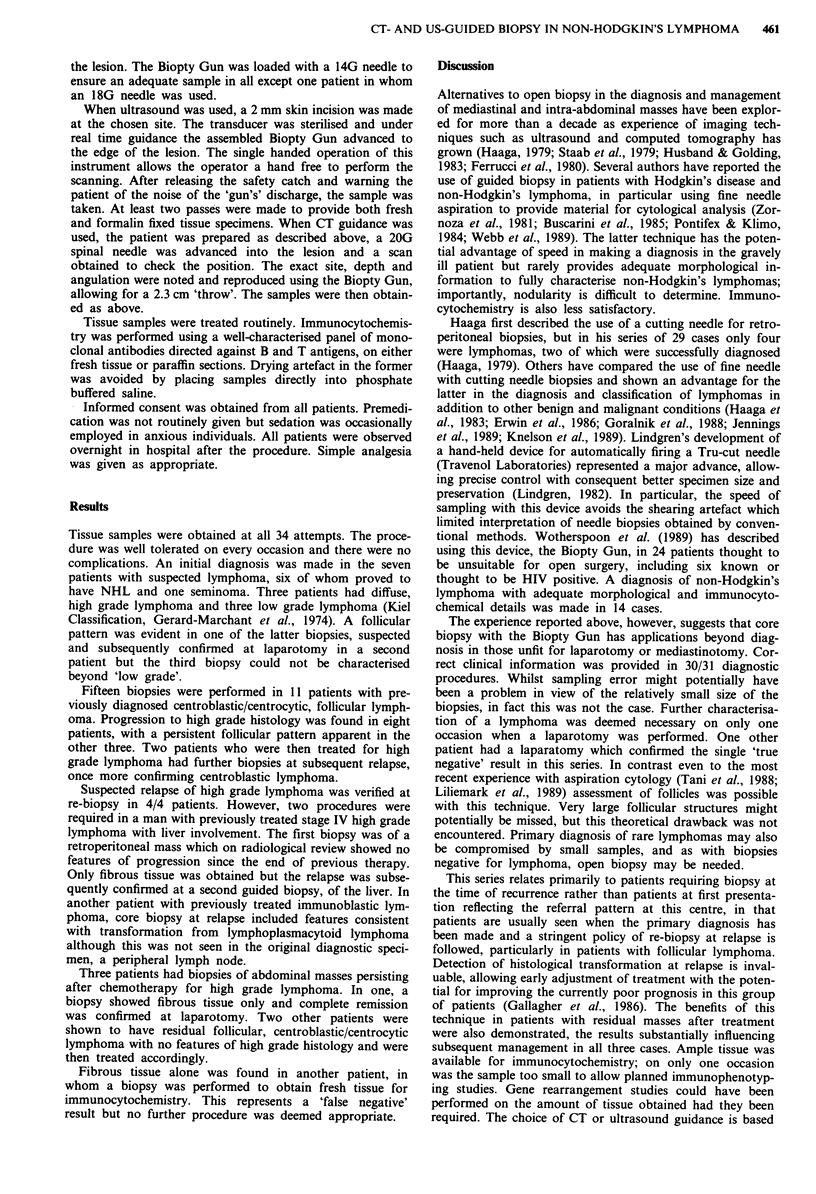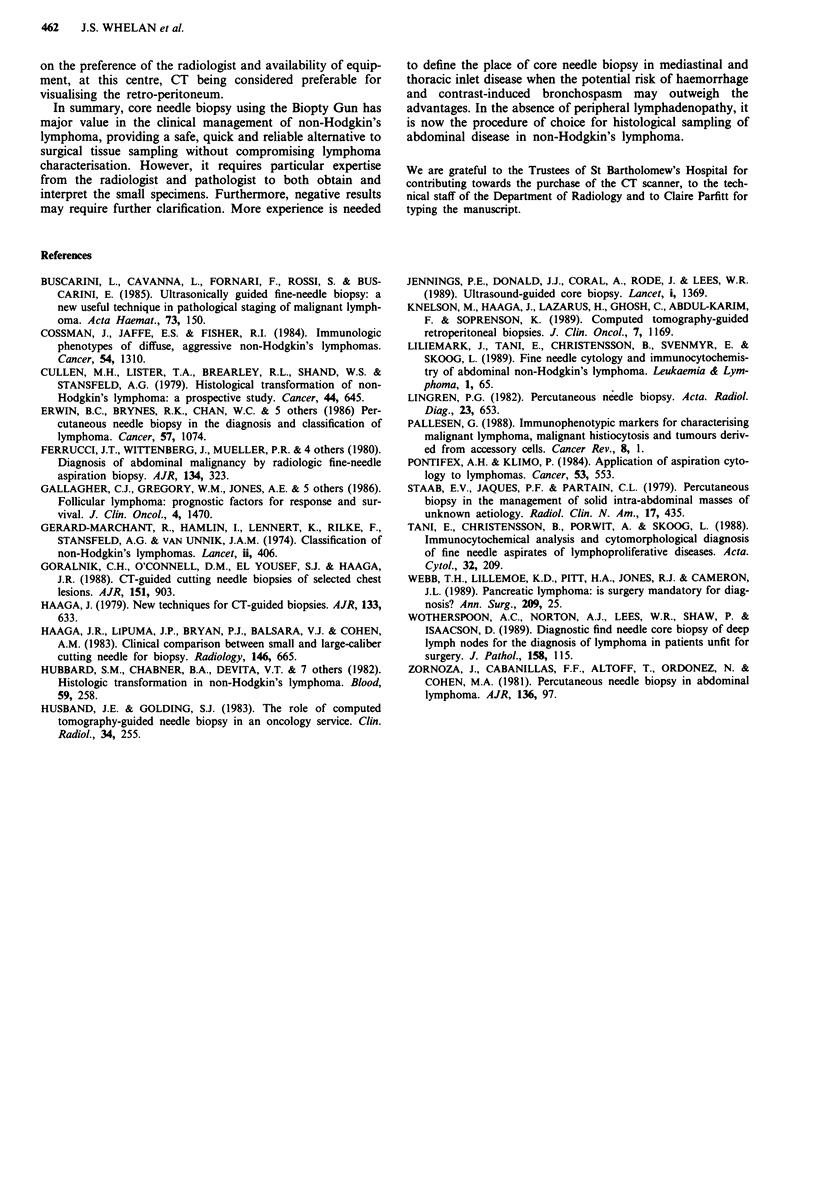# Computed tomography (CT) and ultrasound (US) guided core biopsy in the management of non-Hodgkin's lymphoma.

**DOI:** 10.1038/bjc.1991.107

**Published:** 1991-03

**Authors:** J. S. Whelan, R. H. Reznek, S. J. Daniell, A. J. Norton, T. A. Lister, A. Z. Rohatiner

**Affiliations:** ICRF Department of Medical Oncology, St Bartholomew's Hospital, London, UK.

## Abstract

Histological examination of adequate biopsy specimens is fundamental to the management of patients with non-Hodgkin's lymphoma (NHL). A practical alternative to open biopsy, provided enough tissue can be obtained, has obvious advantages, especially if the lesion in question is deep seated, and might call for laparotomy or thoracotomy. Core biopsy with computed tomography (CT) or ultrasound (US) guidance may be such an alternative, particularly when a spring-loaded firing device is used. Thirty-four biopsies were performed in 26 patients with known or suspected NHL. A primary histological diagnosis was made in 7/7 (six NHL, one seminoma). Relapse was confirmed in 15/15 patients overall. In patients with follicular NHL, 8/15 biopsies showed progression to high grade histology. Biopsies were also performed to assess the nature of residual abnormalities after treatment and to obtain fresh tissue for immunocytochemistry. Tissue was obtained in all cases and a further procedure (two laparotomies, one second needle biopsy) was required on only three occasions. The procedure was well tolerated and there were no complications. This technique is therefore a valuable alternative to more invasive surgical procedures and may be of major benefit in the management of NHL.


Br. J. Cancer (1991), 63, 460-462 © Macmillan Press Ltd., 1991

### Computed tomography (CT) and ultrasound (US) guided core biopsy in the management of non-Hodgkin's lymphoma

J.S. Whelan[1], R.H. Reznek[2], S.J.N. Daniell[2], A.J. Norton[3], T.A. Lister[1] & A.Z.S. Rohatiner[1]

[1]*ICRF Department of Medical Oncology,* [2]*Departments of Radiology and* [3]*Pathology, St Bartholomew's Hospital, London EC1A 7BE, UK.*

**Summary** Histological examination of adequate biopsy specimens is fundamental to the management of patients with non-Hodgkin's lymphoma (NHL). A practical alternative to open biopsy, provided enough tissue can be obtained, has obvious advantages, especially if the lesion in question is deep seated, and might call for laparotomy or thoracotomy. Core biopsy with computed tomography (CT) or ultrasound (US) guidance may be such an alternative, particularly when a spring-loaded firing device is used. Thirty-four biopsies were performed in 26 patients with known or suspected NHL. A primary histological diagnosis was made in 7/7 (six NHL, one seminoma). Relapse was confirmed in 15/15 patients overall. In patients with follicular NHL, 8/15 biopsies showed progression to high grade histology. Biopsies were also performed to assess the nature of residual abnormalities after treatment and to obtain fresh tissue for immunocytochemistry. Tissue was obtained in all cases and a further procedure (two laparotomies, one second needle biopsy) was required on only three occasions. The procedure was well tolerated and there were no complications. This technique is therefore a valuable alternative to more invasive surgical procedures and may be of major benefit in the management of NHL.

Tissue biopsy allowing histological classification is essential for defining the optimal management of patients with non-Hodgkin's lymphoma. It is necessary for establishing a diagnosis, contributing information about natural history, responsiveness to therapy and prognosis. Repeat biopsy may be required to confirm relapse or progression of disease, especially when transformation of follicular lymphoma to high grade histology is suspected (Cullen *et al.*, 1979; Hubbard *et al.*, 1982; Gallagher *et al.*, 1986), and to define the nature of radiological abnormalities remaining after therapy. Immunocytochemical analysis is an important adjunct to morphological diagnosis but requires extra tissue. Furthermore, certain differentiation antigens can only be detected using fresh tissue (Cossman *et al.*, 1984; Pallesen, 1988).

The mainstay of tissue diagnosis in lymphoma is excision biopsy of peripheral lymph nodes. In the absence of palpable disease, resort has been made to more invasive methods such as laparotomy and mediastinotomy but these require general anaesthesia, carry significant morbidity, and are expensive and time consuming. In patients with intra-abdominal and mediastinal disease, guided needle biopsy may offer an alternative. However, in the past, this technique has been limited to providing cytological material only and therefore has been of limited application in lymphoma where morphological accuracy requires more substantial tissue, particularly to determine nodularity (Zornoza *et al.*, 1981; Buscarini *et al.*, 1985; Pontifex & Klimo, 1984; Webb *et al.*, 1989). The use of a spring loaded device to fire a cutting needle has stimulated radiologists to sample otherwise inaccessible tissue and obtain specimens suitable for histologic examination. The use of this device, the Biopty Gun (Radiplast Biopty, Henleys Medical Supplies Ltd, London) was evaluated, at the time of diagnosis or at relapse, in a series of patients with known or suspected non-Hodgkin's lymphoma.

### Patients and methods

### *Patients*

Twenty-six patients in whom radiological evaluation confirmed that appropriate tissue was amenable to biopsy form the basis of this report. Thirteen men and 13 women with a median age of 50 years (range 30-86) had 34 biopsies. The indications for biopsy are shown in Table I.

Seven patients had more than one biopsy. A 66 year old woman with a 12 year history of follicular lymphoma had tissue sampled at three separate relapses. Confirmation of a further relapse was made by second biopsy in three other patients. Fresh tissue for immunophenotyping was needed in two patients, one of whom had had an earlier diagnostic biopsy, the other later requiring clarification of the nature of a residual mass after therapy. An initial biopsy in another patient with suspected relapse of high grade lymphoma garnered fibrous tissue only and a second biopsy was therefore performed.

The platelet count and clotting screen were normal in all patients except one man with a platelet count of $33 \times 10^9 l^{-1}$ who was successfully biopsied under platelet cover.

### *Site and extent of disease biopsied*

A mass had been previously identified in all patients either by computed tomography or ultrasound scanning. The site of disease was intra-abdominal in 27 cases, pelvic in four and intrathoracic in three cases. The intra-abdominal sites included the mesentery in eight and the retroperitoneum in ten. One was intrahepatic. The maximum dimensions ranged from 3-19 cm (approximate volume: 94-1,853 ml).

### *Biopsy technique*

Twenty-eight biopsies were performed under CT (25 abdominal, three thoracic) and six under US guidance. The choice of imaging guidance was based predominantly on likely ease of access for the biopsy. Limited confirmatory scanning was performed in an appropriate position. The skin was then cleaned and sterile towels applied. Local anaesthesia was obtained using 1% lignocaine injected down to the level of

**Table I** Indications for biopsy

| *Indications for biopsy* | *Number of patients* |
|---|---|
| Diagnosis: | 7 |
| Suspected relapse: | |
| Follicular: | 11 |
| High Grade: | 4 |
| Evaluation of residual radiological abnormality | 3 |
| Fresh tissue for immunocytochemistry | 1 |
| Total | 26 |

Correspondence: J. Whelan, ICRF Department of Medical Oncology, St Bartholomew's Hospital, West Smithfield, London EC1A 7BE, UK.
Received 16 August 1990; and in revised form 31 October 1990.

the lesion. The Biopty Gun was loaded with a 14G needle to ensure an adequate sample in all except one patient in whom an 18G needle was used.

When ultrasound was used, a 2 mm skin incision was made at the chosen site. The transducer was sterilised and under real time guidance the assembled Biopty Gun advanced to the edge of the lesion. The single handed operation of this instrument allows the operator a hand free to perform the scanning. After releasing the safety catch and warning the patient of the noise of the 'gun's' discharge, the sample was taken. At least two passes were made to provide both fresh and formalin fixed tissue specimens. When CT guidance was used, the patient was prepared as described above, a 20G spinal needle was advanced into the lesion and a scan obtained to check the position. The exact site, depth and angulation were noted and reproduced using the Biopty Gun, allowing for a 2.3 cm 'throw'. The samples were then obtained as above.

Tissue samples were treated routinely. Immunocytochemistry was performed using a well-characterised panel of monoclonal antibodies directed against B and T antigens, on either fresh tissue or paraffin sections. Drying artefact in the former was avoided by placing samples directly into phosphate buffered saline.

Informed consent was obtained from all patients. Premedication was not routinely given but sedation was occasionally employed in anxious individuals. All patients were observed overnight in hospital after the procedure. Simple analgesia was given as appropriate.

### Results

Tissue samples were obtained at all 34 attempts. The procedure was well tolerated on every occasion and there were no complications. An initial diagnosis was made in the seven patients with suspected lymphoma, six of whom proved to have NHL and one seminoma. Three patients had diffuse, high grade lymphoma and three low grade lymphoma (Kiel Classification, Gerard-Marchant *et al.*, 1974). A follicular pattern was evident in one of the latter biopsies, suspected and subsequently confirmed at laparotomy in a second patient but the third biopsy could not be characterised beyond 'low grade'.

Fifteen biopsies were performed in 11 patients with previously diagnosed centroblastic/centrocytic, follicular lymphoma. Progression to high grade histology was found in eight patients, with a persistent follicular pattern apparent in the other three. Two patients who were then treated for high grade lymphoma had further biopsies at subsequent relapse, once more confirming centroblastic lymphoma.

Suspected relapse of high grade lymphoma was verified at re-biopsy in 4/4 patients. However, two procedures were required in a man with previously treated stage IV high grade lymphoma with liver involvement. The first biopsy was of a retroperitoneal mass which on radiological review showed no features of progression since the end of previous therapy. Only fibrous tissue was obtained but the relapse was subsequently confirmed at a second guided biopsy, of the liver. In another patient with previously treated immunoblastic lymphoma, core biopsy at relapse included features consistent with transformation from lymphoplasmacytoid lymphoma although this was not seen in the original diagnostic specimen, a peripheral lymph node.

Three patients had biopsies of abdominal masses persisting after chemotherapy for high grade lymphoma. In one, a biopsy showed fibrous tissue only and complete remission was confirmed at laparotomy. Two other patients were shown to have residual follicular, centroblastic/centrocytic lymphoma with no features of high grade histology and were then treated accordingly.

Fibrous tissue alone was found in another patient, in whom a biopsy was performed to obtain fresh tissue for immunocytochemistry. This represents a 'false negative' result but no further procedure was deemed appropriate.

### Discussion

Alternatives to open biopsy in the diagnosis and management of mediastinal and intra-abdominal masses have been explored for more than a decade as experience of imaging techniques such as ultrasound and computed tomography has grown (Haaga, 1979; Staab *et al.*, 1979; Husband & Golding, 1983; Ferrucci *et al.*, 1980). Several authors have reported the use of guided biopsy in patients with Hodgkin's disease and non-Hodgkin's lymphoma, in particular using fine needle aspiration to provide material for cytological analysis (Zornoza *et al.*, 1981; Buscarini *et al.*, 1985; Pontifex & Klimo, 1984; Webb *et al.*, 1989). The latter technique has the potential advantage of speed in making a diagnosis in the gravely ill patient but rarely provides adequate morphological information to fully characterise non-Hodgkin's lymphomas; importantly, nodularity is difficult to determine. Immunocytochemistry is also less satisfactory.

Haaga first described the use of a cutting needle for retroperitoneal biopsies, but in his series of 29 cases only four were lymphomas, two of which were successfully diagnosed (Haaga, 1979). Others have compared the use of fine needle with cutting needle biopsies and shown an advantage for the latter in the diagnosis and classification of lymphomas in addition to other benign and malignant conditions (Haaga *et al.*, 1983; Erwin *et al.*, 1986; Goralnik *et al.*, 1988; Jennings *et al.*, 1989; Knelson *et al.*, 1989). Lindgren's development of a hand-held device for automatically firing a Tru-cut needle (Travenol Laboratories) represented a major advance, allowing precise control with consequent better specimen size and preservation (Lindgren, 1982). In particular, the speed of sampling with this device avoids the shearing artefact which limited interpretation of needle biopsies obtained by conventional methods. Wotherspoon *et al.* (1989) has described using this device, the Biopty Gun, in 24 patients thought to be unsuitable for open surgery, including six known or thought to be HIV positive. A diagnosis of non-Hodgkin's lymphoma with adequate morphological and immunocytochemical details was made in 14 cases.

The experience reported above, however, suggests that core biopsy with the Biopty Gun has applications beyond diagnosis in those unfit for laparotomy or mediastinotomy. Correct clinical information was provided in 30/31 diagnostic procedures. Whilst sampling error might potentially have been a problem in view of the relatively small size of the biopsies, in fact this was not the case. Further characterisation of a lymphoma was deemed necessary on only one occasion when a laparotomy was performed. One other patient had a laparatomy which confirmed the single 'true negative' result in this series. In contrast even to the most recent experience with aspiration cytology (Tani *et al.*, 1988; Liliemark *et al.*, 1989) assessment of follicles was possible with this technique. Very large follicular structures might potentially be missed, but this theoretical drawback was not encountered. Primary diagnosis of rare lymphomas may also be compromised by small samples, and as with biopsies negative for lymphoma, open biopsy may be needed.

This series relates primarily to patients requiring biopsy at the time of recurrence rather than patients at first presentation reflecting the referral pattern at this centre, in that patients are usually seen when the primary diagnosis has been made and a stringent policy of re-biopsy at relapse is followed, particularly in patients with follicular lymphoma. Detection of histological transformation at relapse is invaluable, allowing early adjustment of treatment with the potential for improving the currently poor prognosis in this group of patients (Gallagher *et al.*, 1986). The benefits of this technique in patients with residual masses after treatment were also demonstrated, the results substantially influencing subsequent management in all three cases. Ample tissue was available for immunocytochemistry; on only one occasion was the sample too small to allow planned immunophenotyping studies. Gene rearrangement studies could have been performed on the amount of tissue obtained had they been required. The choice of CT or ultrasound guidance is based on the preference of the radiologist and availability of equipment, at this centre, CT being considered preferable for visualising the retro-peritoneum.

In summary, core needle biopsy using the Biopty Gun has major value in the clinical management of non-Hodgkin's lymphoma, providing a safe, quick and reliable alternative to surgical tissue sampling without compromising lymphoma characterisation. However, it requires particular expertise from the radiologist and pathologist to both obtain and interpret the small specimens. Furthermore, negative results may require further clarification. More experience is needed to define the place of core needle biopsy in mediastinal and thoracic inlet disease when the potential risk of haemorrhage and contrast-induced bronchospasm may outweigh the advantages. In the absence of peripheral lymphadenopathy, it is now the procedure of choice for histological sampling of abdominal disease in non-Hodgkin's lymphoma.

We are grateful to the Trustees of St Bartholomew's Hospital for contributing towards the purchase of the CT scanner, to the technical staff of the Department of Radiology and to Claire Parfitt for typing the manuscript.

### References

BUSCARINI, L., CAVANNA, L., FORNARI, F., ROSSI, S. & BUSCARINI, E. (1985). Ultrasonically guided fine-needle biopsy: a new useful technique in pathological staging of malignant lymphoma. *Acta Haemat.*, **73**, 150.

COSSMAN, J., JAFFE, E.S. & FISHER, R.I. (1984). Immunologic phenotypes of diffuse, aggressive non-Hodgkin's lymphomas. *Cancer*, **54**, 1310.

CULLEN, M.H., LISTER, T.A., BREARLEY, R.L., SHAND, W.S. & STANSFELD, A.G. (1979). Histological transformation of non-Hodgkin's lymphoma: a prospective study. *Cancer*, **44**, 645.

ERWIN, B.C., BRYNES, R.K., CHAN, W.C. & 5 others (1986) Percutaneous needle biopsy in the diagnosis and classification of lymphoma. *Cancer*, **57**, 1074.

FERRUCCI, J.T., WITTENBERG, J., MUELLER, P.R. & 4 others (1980). Diagnosis of abdominal malignancy by radiologic fine-needle aspiration biopsy. *AJR*, **134**, 323.

GALLAGHER, C.J., GREGORY, W.M., JONES, A.E. & 5 others (1986). Follicular lymphoma: prognostic factors for response and survival. *J. Clin. Oncol.*, **4**, 1470.

GERARD-MARCHANT, R., HAMLIN, I., LENNERT, K., RILKE, F., STANSFELD, A.G. & VAN UNNIK, J.A.M. (1974). Classification of non-Hodgkin's lymphomas. *Lancet*, **ii**, 406.

GORALNIK, C.H., O'CONNELL, D.M., EL YOUSEF, S.J. & HAAGA, J.R. (1988). CT-guided cutting needle biopsies of selected chest lesions. *AJR*, **151**, 903.

HAAGA, J. (1979). New techniques for CT-guided biopsies. *AJR*, **133**, 633.

HAAGA, J.R., LIPUMA, J.P., BRYAN, P.J., BALSARA, V.J. & COHEN, A.M. (1983). Clinical comparison between small and large-caliber cutting needle for biopsy. *Radiology*, **146**, 665.

HUBBARD, S.M., CHABNER, B.A., DEVITA, V.T. & 7 others (1982). Histologic transformation in non-Hodgkin's lymphoma. *Blood*, **59**, 258.

HUSBAND, J.E. & GOLDING, S.J. (1983). The role of computed tomography-guided needle biopsy in an oncology service. *Clin. Radiol.*, **34**, 255.

JENNINGS, P.E., DONALD, J.J., CORAL, A., RODE, J. & LEES, W.R. (1989). Ultrasound-guided core biopsy. *Lancet*, **i**, 1369.

KNELSON, M., HAAGA, J., LAZARUS, H., GHOSH, C., ABDUL-KARIM, F. & SOPRENSON, K. (1989). Computed tomography-guided retroperitoneal biopsies. *J. Clin. Oncol.*, **7**, 1169.

LILIEMARK, J., TANI, E., CHRISTENSSON, B., SVENMYR, E. & SKOOG, L. (1989). Fine needle cytology and immunocytochemistry of abdominal non-Hodgkin's lymphoma. *Leukaemia & Lymphoma*, **1**, 65.

LINGREN, P.G. (1982). Percutaneous needle biopsy. *Acta. Radiol. Diag.*, **23**, 653.

PALLESEN, G. (1988). Immunophenotypic markers for characterising malignant lymphoma, malignant histiocytosis and tumours derived from accessory cells. *Cancer Rev.*, **8**, 1.

PONTIFEX, A.H. & KLIMO, P. (1984). Application of aspiration cytology to lymphomas. *Cancer*, **53**, 553.

STAAB, E.V., JAQUES, P.F. & PARTAIN, C.L. (1979). Percutaneous biopsy in the management of solid intra-abdominal masses of unknown aetiology. *Radiol. Clin. N. Am.*, **17**, 435.

TANI, E., CHRISTENSSON, B., PORWIT, A. & SKOOG, L. (1988). Immunocytochemical analysis and cytomorphological diagnosis of fine needle aspirates of lymphoproliferative diseases. *Acta. Cytol.*, **32**, 209.

WEBB, T.H., LILLEMOE, K.D., PITT, H.A., JONES, R.J. & CAMERON, J.L. (1989). Pancreatic lymphoma: is surgery mandatory for diagnosis? *Ann. Surg.*, **209**, 25.

WOTHERSPOON, A.C., NORTON, A.J., LEES, W.R., SHAW, P. & ISAACSON, D. (1989). Diagnostic find needle core biopsy of deep lymph nodes for the diagnosis of lymphoma in patients unfit for surgery. *J. Pathol.*, **158**, 115.

ZORNOZA, J., CABANILLAS, F.F., ALTOFF, T., ORDONEZ, N. & COHEN, M.A. (1981). Percutaneous needle biopsy in abdominal lymphoma. *AJR*, **136**, 97.